# Thoracic Ultrasound in Assessment of the Pleura in Connective Tissue Disease

**DOI:** 10.5152/ArchRheumatol.2025.25055

**Published:** 2025-12-01

**Authors:** Serap Diktaş Tahtasakal, Coşkun Doğan

**Affiliations:** 1 Department of Pulmonology, İstanbul Fatih Sultan Mehmet Training and Research Hospital, İstanbul, Türkiye; 2 Department of Pulmonology, İstanbul Medeniyet University Faculty of Medicine, İstanbul, Türkiye

**Keywords:** Connective tissue disease, pleura, pleural thickness, thoracic ultrasound

## Abstract

**Background/Aims:**

To investigate the use of thoracic ultrasound (US) in the assessment of the pleura in connective tissue disease (CTD).

**Materials and Methods:**

The clinical, radiologic, and demographic data were recorded from patients who were diagnosed as having CTD at least 1 year before the study. Thoracic US was performed for pleural assessments in all patients who met the inclusion criteria. The thickness of the parietal and visceral layers of the pleura and pleural space was measured in millimeters and recorded in both the CTD and healthy control groups, and data from the CTD group were compared with those from the healthy control group.

**Results:**

A total of 86 participants, 44 (51.2%) with CTD and 42 (48.8%) healthy volunteers, were included in the study. There were 37 (84.1%) females with a mean age of 55.1 ± 11.1 years in the CTD group and 23 (54.8%) females with a mean age of 56 ± 11.8 years in the control group. In the CTD group, the mean parietal pleural thickness, visceral pleural thickness, and pleural space were 0.61 ± 0.16 mm, 0.61 ± 0.17 mm, and 0.77 ± 0.5 mm, respectively, and in the control group, these values were 0.42 ± 0.08 mm, 0.44 ± 0.09 mm, and 0.47 ± 0.15 mm, respectively (*P* < .001).

**Conclusion:**

Pleural thickness was increased in patients with CTD compared with healthy controls. The lungs are among the target organs in CTD, and US is a non-invasive and radiation-free imaging modality that can be used in the assessment of pleural involvement.

**Main Points**

- Thoracic ultrasound showed increased parietal and visceral pleural thickness and pleural space measurements in patients with connective tissue disease compared with healthy controls.
- Ultrasonography can be used to assess pleural involvement in connective tissue diseases.
- Pleural thickening was observed in all connective tissue disease subgroups.
- Thoracic ultrasound identified pleural changes that were not evident in routine radiologic evaluations.
- Thoracic ultrasound offers a non-invasive, radiation-free method for evaluating pleural involvement in connective tissue diseases.

## Introduction

Currently, thoracic ultrasound (US) is increasingly used in the clinical practice of pulmonology medicine due to many reasons, including its usability for the diagnosis of several pleural-pulmonary pathologic conditions, feasibility for real-time interventions, lack of exposure to radiation for both the patient and operator, non-invasiveness, affordability, and the ability to be performed at the bedside when needed.^1^ Ultrasound devices were invented in the 1920s, but they have been in use as a diagnostic tool since the 1940s. At that time, it was considered that the lungs, being air-filled organs, could not reflect sound waves emitted by a transducer to create an image on the monitor, and therefore, US was not included in the diagnostic examination for parenchymal diseases of the lung, in particular. However, such a disadvantage does not exist in imaging of the pleura and pleural effusions; therefore, this became its first use in the field of pulmonology.^2^

Connective tissue disease (CTD) represents a group of heterogeneous, multisystem, autoimmune diseases characterized by chronic inflammation and circulating antibodies leading to organ damage. The inflammation targets the extracellular matrix, which is a structural support for the organs in the body and is mainly made up of collagen and elastin. Connective tissue disease can severely impact the lungs due to their dense connective tissue content and rich blood supply.^3,4^ Although the pathophysiology of the pleural disease/involvement in CTD is poorly understood, endothelial injury is thought to be related to the activation of the complement system by circulating immune complexes deposited in the pleura.^5^

The pleura may be affected/involved in almost all forms of CTD, but most notably in systemic lupus erythematosus (SLE) and rheumatoid arthritis (RA). Although a number of studies have investigated parenchymal involvement using US in CTD, there are very limited studies on the use of US to assess pleural involvement in the disease.^6-8^ In this study, thoracic US was used to assess pleural thickness and compare findings from patients with CTD with those of healthy controls to see potential differences.

## Methods

### Patient Population

This study included patients with CTD attending İstanbul Fatih Sultan Mehme Training and Research Hospital, Department of Pulmonologyt Clinics between May 2023 and June 2024 for an assessment before starting therapy with biologic agents. The study was designed as a prospective study and was conducted in line with the principles of the Declaration of Helsinki. The exclusion criteria were the absence of a diagnosis of definite CTD or a diagnosis of CTD made less than 1 year ago. The exclusion and inclusion criteria are presented in Table 1. The study was approved by the Ethics Review Board of İstanbul Medeniyet University Faculty of Medicine, Göztepe Prof. Dr. Süleyman Yalçın City Hospital (board decision number 2023/0463; date: July 26, 2023). Written informed consent was obtained from the patients.

In the clinic, the assessment for use of biologic agents in patients with CTD included a detailed medical history, physical examination, pulmonary function test (PFT), chest X-rays [P-A (posterior–anterior) view], and computed tomography (CT) scans of the chest, if needed. For each patient meeting the inclusion criteria, clinical, imaging (chest X-rays and chest CT scans, when needed), and demographic data were recorded. All patients who provided a signed informed consent form and met the inclusion criteria were assessed using thoracic US by a pulmonologist (C.D.) experienced in thoracic US. Sonographic measurements of the pleural thickness and the distance between the 2 layers of the pleura were taken and noted for all patients.

### Thoracic Ultrasound in the Assessment of the Pleura

**Figure 1.**
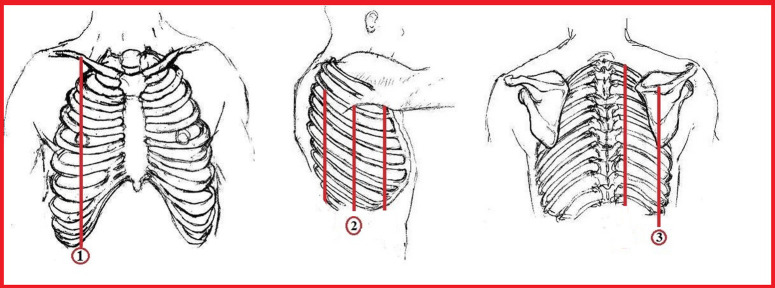
Pre-specified anatomic lines (1: Linea mid-clavicularis, 2: Linea axillaris media, 3: Linea scapularis).

Ultrasound examinations were performed while the patient was in a sitting position, by moving the transducer transversally and longitudinally in the intercostal spaces, across the entire thorax along the predetermined anatomic lines in both hemithorax including the midclavicular, midaxillary, and medial scapular lines using a GE Logiq P9 ultrasound system with a linear array transducer at 10 MHz on superficial mode, and the area of maximum pleural thickness was determined (Figure 1).

**Figure 2.**
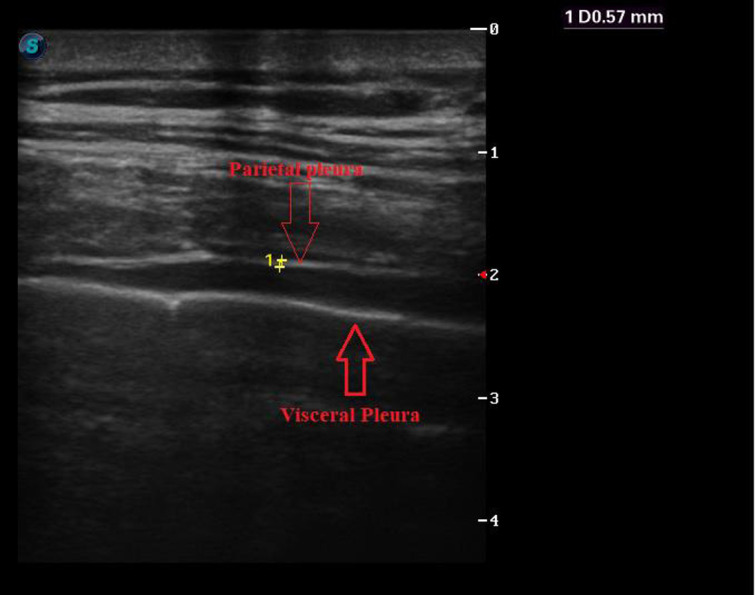
Ultrasonographic Imaging of the parietal and visceral pleura using a linear array transducer at 10 MHz on superficial mode (visceral and parietal pleura are indicated with red arrows).

The visceral pleura, the inner layer covering the lungs, is farther from the linear probe and the outer layer. The parietal pleura is characterized by a fine, bright echogenic line just above the physiologic pleural fluid contained between these 2 layers (Figure 2). The thickness of the visceral and parietal layers and the space between these layers (pleural space [PS]) were measured and noted in millimeters.

### Statistical Analysis

Descriptive statistics are presented as mean and SD for continuous variables and as frequency and percentage for categorical variables. The Shapiro–Wilk test was used to assess the normality of distribution for continuous variables. For comparisons between CTD and healthy control groups, the Independent Samples *t*-test was applied to normally distributed continuous variables, while the Mann–Whitney *U-*test was used for non-normally distributed variables. The chi-square test was applied to compare proportions of categorical variables. The Kruskal–Wallis test was used to compare 3 or more independent groups, and a post hoc analysis was performed when necessary. A *P*-value of <.05 was considered statistically significant in all analyses. Statistical analyses were performed using IBM SPSS (Version 21) (IBM SPSS Corp.; Armonk, NY, USA).

## Results

The study was conducted on 86 participants, including 44 patients with CTD (51.2%) [n = 37 (84.1%) females and 7 (15.9%) males] and 42 healthy controls (48.8%) [n = 23 (54.8%) females and 19 males (45.2%)]. There was no statistically significant difference between the groups in age (*P* = .520), whereas statistically significant differences were observed in sex (*P* = .003).

In the CTD group, 18 (40.9%) patients had a history of smoking with a mean consumption of 6.9 ± 10.9 pack-years. In the control group, 21 (50%) patients had a history of smoking with a mean consumption of 12.5 ± 19.5 pack-years (*P* = .397 and *P* = .284, respectively).

**Figure 3.**
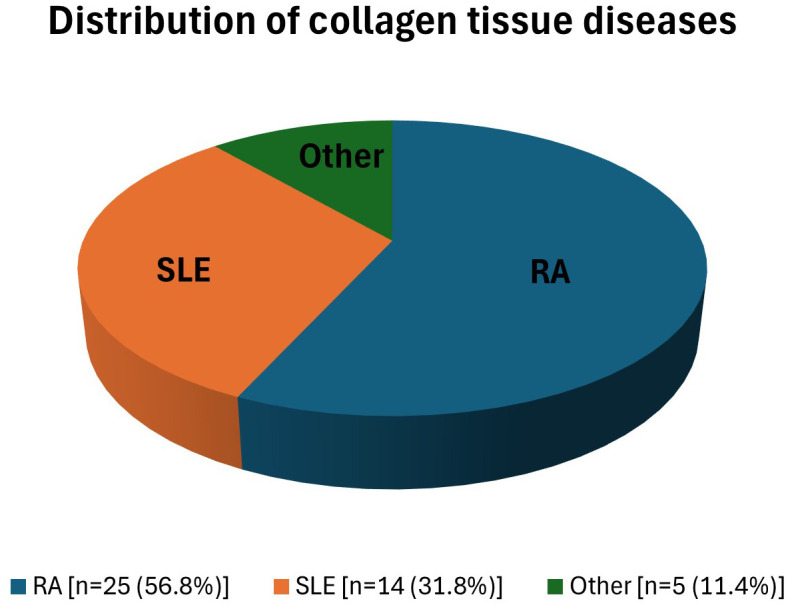
Distribution of collagen tissue diseases.

The most common disease was RA (n = 25, 56.8%) (Figure 3). The mean CTD duration was 13.4 ± 8.19 years. In the CTD group, 26 (59.1%) patients had no chronic disease other than CTD; 18 (40.9%) patients reported comorbidities. The most common comorbidity was hypertension (n = 17, 38.6%). Four (9.1%) patients from the CTD group presented with respiratory symptoms; 40 (90.0%) patients with CTD had no respiratory symptoms. In the CTD group, the physical exam revealed abnormal findings in 2 (4.5%) patients; physical exams were normal in 42 (95.5%) patients. Abnormal findings were detected in P-A chest X-rays of 4 patients; no pathological findings were seen in 40 (90.9%) patients in the CTD group. All patients in the CTD group underwent chest CT scans; 33 (75%) patients had no abnormal findings, and 11 (25%) had abnormal findings. Pleural thickness was not observed as a CT finding in any of them. Detailed clinical, radiologic, and demographic data from the CTD group are presented in Table 1. No chronic diseases or pathological, physical, or imaging findings were detected in the control group.

No statistically significant differences were found between the CTD group and control group in terms of force vital capacity (FVC%), forced expiratory volume in 1 second (FEV1%), and FEV1/FVC values in PFT (*P* = .454, *P* = .526, and *P* = .152, respectively) (Table 2).

The mean parietal pleural thickness (PPT), the mean visceral pleural thickness (VPT), and PS were 0.61 ± 0.16 mm, 0.61 ± 0.17 mm, and 0.77 ± 0.5 mm in the CTD group, and 0.42 ± 0.08 mm, 0.44 ± 0.09 mm, and 0.47 ± 0.15 mm in the control group, respectively (*P* < .001) (Table 3).

The CTD group was stratified based on primary disease, and the PT (pleural thickness) and PS (pleural space) were statistically significantly increased in all primary disease subgroups compared with the control group (*P* < .05) (Table 4). Intragroup comparisons in the CTD group revealed no statistically significant differences among the primary disease subgroups in PPT, VPT, and PS measurements (*P* = .811, *P* = .518, and *P* = .142) (Table 5).

## Discussion

The impact of CTD on the lungs has long been known. The lung interstitium, respiratory airways, pleura, and vessels are the most common targets in circulating autoantibody-mediated conditions that are generally characterized by inflammation, tissue damage, and abnormal repair processes. To date, many studies have been conducted to investigate the impact of CTD on the lungs, based on signs and symptoms, diagnostic imaging (chest PA [Posterio-Anterior] X-rays, chest CT, and high-resolution computed tomography [HRCT]) findings, and changes in PFTs and diffusion tests, and the results have been well defined. In this study, thoracic US was used to investigate the impact of CTD on the pleura and potential differences between patients with CTD and healthy controls in sonographic features. It was concluded that the thickness of the pleural layers and PS were increased in the CTD group.

Using a high-frequency transducer (usually at 7.5-12 MHz) with the longitudinal axis placed parallel to the ribs, the pleura can be seen as 2 parallel echogenic lines 2-3 mm in thickness.^9-11^ There are many advantages to using US to examine the pleura, including lack of exposure to radiation for both patient and operator, ease of use, widespread availability, robust diagnostic utility, high safety, and affordability. Therefore, the US has been more widely used than other conventional imaging methods.^12^ In 1992, Yang et al^13^ demonstrated that if the pleural thickness was ≥3 mm, the probability of the presence of any disease that might lead to exudative pleural effusion was 96.1%. Following this study, many studies have been conducted to investigate the use of US in the assessment of the pleura and to detect pleural diseases, demonstrating links between pleural thickening and various diseases. Most of these studies revealed that pleural thickening was associated with exudative pleural effusions and malignant pleural effusions.^14-16^

Rheumatoid arthritis and SLE are the 2 diseases that are most associated with pleural involvement in CTD.^17^ The most prevalent pleural manifestations of RA include pleural effusions, acute fibrous pleuritis, chronic fibrous pleuritis, and pleural adhesions.^18,19^ In RA, the pleura becomes infiltrated by inflammatory cells. Postmortem studies in RA have indicated that the prevalence of pleural involvement has been underestimated.^17,20^ Pleuritis is among the most common pulmonary manifestations in SLE. Pleural involvement forms part of the spectrum of serosal diseases in SLE, and other serosal membranes (e.g., pericarditis, peritoneum) may also become involved. Microscopic features of pleuritis may include the accumulation of fibrin in the pleura, fibrosis, and non-specific inflammation of the pleura. In parallel with RA, postmortem studies in SLE have also demonstrated that the prevalence of pleural involvement has been underestimated, and pleural disease may be present in about 93% of patients with SLE.^21-23^ In these 2 diseases, pleural involvement can occur through different mechanisms, but ultimately, fibro-inflammatory lesions can result in pleural thickening in both diseases.^22^

A number of studies investigated the use of US in CTD. Most studies focused on sonographic detection of parenchymal involvement in CTD. Many previous studies documented the high sensitivity and specificity of the presence of multiple B lines detected using thoracic US in interstitial lung disease (ILD) associated with CTD.^24-26^

In one of the few studies on the use of US in the assessment of pleural involvement in CTD, Moazedi-Fuerst et al^27^ compared findings from 45 patients with CTD with those of 40 healthy controls and reported pleural thickening in 95% of patients with CTD, particularly in those with interstitial involvement (*P* < .001). This was an important study because it suggested the use of US for assessing the pleura in CTD. In reference to the ultrasonographic detection of increased PT detected in this study, an article^28^ on the value of chest CT scans and transthoracic lung US for patients with systemic sclerosis was published in 2022. The article^28^ included a consensus statement by Austrian rheumatologists, pneumologists, and radiologists, and pleural thickening (>3 mm) was defined as an important sonographic finding. Based on the consensus achieved by the authors of this article, transthoracic lung US should be repeated annually, and PFTs should be repeated every 6-12 months in asymptomatic patients if chest CT scans at diagnosis do not reveal any abnormal findings.^28^ This current opinion is important because it suggests the use of US for the assessment of the pleura in CTD. Pinal-Fernandez et al^29^ investigated potential associations between pleural irregularity and ILD in patients with systemic sclerosis and antisynthetase syndrome and reported significantly higher rates of pleural irregularity in patients who developed ILD (35.3% vs. 6%, *P* < .001). Unlike the current study, that study was not focused on pleural thickness; however, it is important because it supports the use of US in CTD.^29^

This study has some limitations that should be considered when interpreting the data or planning future studies. The results cannot be generalized due to the limited sample size from a single center. The performance of pleural imaging by a single operator may be considered an important disadvantage because any potential interrater differences remain unknown.

In conclusion, the US is believed to have many advantages in detecting several lung diseases and may be an alternative to conventional imaging methods (chest CT) for the assessment of the pleura in CTD. Future studies comparing chest CT scans and thoracic US will determine which will be the gold standard diagnostic modality.

## Figures and Tables

**Table 1.** Clinical, Demographic, Laboratory, and Treatment Characteristics of the Cases

	CTD Group (n = 44)	Control Group (n = 42)	*P*
Age (mean ± SD) (years)	55.1 ± 11.1	56 ± 11.8	.520
Gender, n (%) (female/male)	37 (84.1)/7 (15.9)	23 (54.8)/19 (45.2)	.003
History of smoking, n (%)	18 (40.9)	21 (50)	.397
History of smoking (pack-year) (mean ± SD)	6.9 ± 10.9	12.5 ± 19.5	.284
CTD, n (%)			
RA	25 (56.8)	–	
SLE	14 (31.8)	–	
Other	5 (11.4)	–	
Comorbidities, n (%)			
Comorbidities	18 (40.9)	–	
Hypertension	17 (38.6)	–	
Diabetes mellitus	9 (20.5)	–	
CRD	9 (20.5)	–	
CHD	8 (18.2)	–	
Cancer	3 (6.8)	–	
CND	2 (4.5)	–	
CKD	1 (2.3)	–	
Symptoms, n (%)			
Respiratory symptoms	4 (9.1)	–	
Shortness of breath	3 (6.8)	–	
Coughing	2 (4.5)	–	
Sputum	1 (2.3)	–	
Wheezing	1 (2.3)	–	
Chest pain	1 (2.3)	–	
Physical exam findings, n (%)			
Physical exam (normal/abnormal)	40 (90.9)/4 (9.1)	–	
Rales	1 (2.3	–	
Rhonchi	1 (2.3	–	
Decreased respiratory sounds	–	–	
Radiological Features, n (%)			
Chest X-rays (normal/abnormal)	40 (90.9/4 (1)	–	
Pleural effusion	–	–	
Consolidacion	–	–	
Atelectasis	–	–	
Pleural thickness	–	–	
Increased ventilation	2 (4.5)	–	
Fibrosis	2 (4.5)	–	
Thorax CT (normal/abnormal), n (%)	33 (75)/11 (25)	–	
Pleural effusion	1 (2.3)	–	
Parenchymal sequelae	3 (6.8)	–	
Atelectasis	1 (2.3)	–	
Hyperinflation	1 (2.3)	–	
Apical fibrosis/sequelae	5 (11.4)	–	

CHD, chronic heart disease; Chest X-rays, P-A (posterior–anterior) view; CKD, chronic kidney disease; CND, chronic neurologic disease; CRD, chronic respiratory disease; CT, computed tomography; CTD, connective tissue disease; RA, rheumatoid arthritis; SLE, systemic lupus erythematosus.

**Table 2.** Pulmonary Function Test Results of the Cases

PFT Parameters	CTD Group (n = 44)	Control Group (n = 42)	*P*
FEV1/FVC (mean ± SD)	84.6 ± 7.6	81.8 ± 5.5	.152
FEV1 Lt (mean ± SD)	2.18 ± 0.49	2.27 ± 0.73	.539
FEV1% (mean ± SD)	88.6 ± 16.9	85.8 ± 16.9	.526
FVC Lt (mean ± SD)	2.61 ± 0.61	2.73 ± 0.85	.824
FVC % (mean ± SD)	89.1 ± 17.5	85.9 ± 15.9	.454

CTD, connective tissue disease; FEV1, force expiratory volume in 1st second; FVC, force vital capacity; Lt, liter; PFT, pulmonary function test.

**Table 3.** Thoracic Ultrasonographic Measurements of the Cases

Thoracic Ultrasonographic Measurements	CTD Group (n = 44)	Control Group (n = 42)	*P*
Right midclavicular VPT (mm) (mean ± SD)	0.61 ± 0.17	0.44 ± 0.13	<.001
Right midclavicular PPT (mm) (mean ± SD)	0.63 ± 0.18	0.41 ± 0.1	<.001
Right midclavicular PS (mm) (mean ± SD)	0.63 ± 0.23	0.46 ± 0.13	<.001
Right axillary VPT (mm) (mean ± SD)	0.57 ± 0.17	0.46 ± 0.12	<.001
Right axillary PPT(mm) (mean ± SD)	0.56 ± 0.17	0.42 ± 0.09	<.001
Right axillary PS (mm) (mean ± SD)	0.6 ± 0.20	0.44 ± 0.12	<.001
Right scapular VPT (mm) (mean ± SD)	0.60 ± 0.22	0.44 ± 0.12	<.001
Right scapular PPT (mm) (mean ± SD)	0.59 ± 0.18	0.44 ± 0.12	<.001
Right scapular PS (mm) (mean ± SD)	0.58 ± 0.27	0.44 ± 0.11	.005
Left midclavicular VPT(mm) (mean ± SD)	0.63 ± 0.26	0.45 ± 0.12	<.001
Left midclavicular PPT (mm) (mean ± SD)	0.64 ± 0.27	0.44 ± 0.11	<.001
Left midclavicular PS (mm) (mean ± SD)	0.81 ± 0.99	0.59 ± 0.71	<.001
Left axillary VPT (mm) (mean ± SD)	0.67 ± 0.23	0.41 ± 0.1	<.001
Left axillary PPT (mm) (mean ± SD)	0.62 ± 0.26	0.39 ± 0.1	<.001
Left axillary PS (mm) (mean ± SD)	1.21 ± 1.68	0.44 ± 0.15	<.001
Left scapular VPT (mm) (mean ± SD)	0.61 ± 0.2	0.43 ± 0.11	<.001
Left scapular PPT (mm) (mean ± SD)	0.59 ± 0.19	0.42 ± 0.12	<.001
Left scapular PS (mm) (mean ± SD)	0.82 ± 0.79	0.45 ± 0.15	<.001
Mean PPT (mm) (mean ± SD)	0.61 ± 0.16	0.42 ± 0.08	<.001
Mean VPT (mm) (mean ± SD)	0.61 ± 0.17	0.44 ± 0.09	<.001
Mean PS (mm) (mean ± SD)	0.77 ± 0.5	0.47 ± 0.15	<.001

CTD, connective tissue disease; mm, milimeter; PPT, parietal pleural thickness; PS, pleural space; VPT, visceral pleural thickness.

**Table 4.** Distribution of Pleural Thickness According to Connective Tissue Disease

Primary CTD	CTD PPT (mm) (mean ± SD)	Control Group PPT (mm) (mean ± SD)	*P*
RA (n = 25)	0.62 ± 0.19	0.42 ± 0.08	<.001
SLE (n = 14)	0.60 ± 0.09	0.42 ± 0.08	<.001
Other (n = 5)	0.62 ± 0.19	0.42 ± 0.08	<.001
**Primary CTD**	**CTD VPT** (mm) (mean ± SD)	**Control Group VPT** (mm) (mean ± SD)	***P***
RA (n = 25)	0.61 ± 0.19	0.44 ± 0.09	<.001
SLE (n = 14)	0.64 ± 0.13	0.44 ± 0.09	<.001
Other (n = 5)	0.64 ± 0.08	0.44 ± 0.09	<.001
**Primary CTD**	**CTD PS** (mm) (mean ± SD)	**Control Group PS** (mm) (mean ± SD)	***P***
RA (n = 25)	0.92 ± 0.61	0.47 ± 0.15	<.001
SLE (n = 14)	0.56 ± 0.11	0.47 ± 0.15	.010
Other (n = 5)	0.66 ± 0.07	0.47 ± 0.15	.001

CTD, connective tissue disease; mm, milimeter; PPT, parietal pleural thickness; PS, pleural space; RA, rheumatoid arthritis; SLE, systemic lupus erythematosus; VPT, visceral pleural thickness.

**Table 5.** Connective Tissue Disease Comparison of Pleural Thicknesses with Each Other

	RA	SLE	Other	*P*
PPT (mm) (mean ± SD)	0.62 ± 0.19	0.60 ± 0.09	0.62 ± 0.19	.811
VPT (mm) (mean ± SD)	0.61 ± 0.19	0.64 ± 0.13	0.64 ± 0.08	.518
PS (mm) (mean ± SD)	0.92 ± 0.61	0.56 ± 0.11	0.66 ± 0.07	.142

PPT, parietal pleural thickness; PS, pleural space; RA, rheumatoid arthritis; SLE, systemic lupus erythematosus; VPT, visceral pleural thickness.
